# The Remaining Useful Life Prediction Method of a Hydraulic Pump under Unknown Degradation Model with Limited Data

**DOI:** 10.3390/s23135931

**Published:** 2023-06-26

**Authors:** Fenghe Wu, Jun Tang, Zhanpeng Jiang, Yingbing Sun, Zhen Chen, Baosu Guo

**Affiliations:** 1 Department of Mechanical Engineering, Yanshan University, Qinhuangdao 066004, China; risingwu@ysu.edu.cn (F.W.); jun.tang@stumail.ysu.edu.cn (J.T.); jzp@stumail.ysu.edu.cn (Z.J.); chenzhen@stumail.ysu.edu.cn (Z.C.); guobaosu@ysu.edu.cn (B.G.); 2 Heavy-Duty Intelligent Manufacturing Equipment Innovation Center of Hebei Province, Yanshan University, Qinhuangdao 066004, China

**Keywords:** pumps, remaining useful life, reliability, degradation model, predictive maintenance

## Abstract

This study proposes a remaining useful life (RUL) prediction method using limited degradation data with an unknown degradation model for hydraulic pumps with long service lives and no failure data in turbine control systems. The volumetric efficiency is calculated based on real-time monitoring signal data, and it is used as the degradation indicator. The optimal degradation curve is established using the degradation trajectory model, and the optimal probability distribution model is selected via the K-S test. The above process was repeated to optimize the degradation model and update parameters in different performance degradation stages of the hydraulic pump, providing quantification of the prediction uncertainty and enabling accurate online prediction of the hydraulic pump’s RUL. Finally, an RUL test bench for hydraulic pumps is built for verification. The results show that the proposed method is convenient, efficient, and has low model complexity. The method enables online accurate prediction of the RUL of hydraulic pumps using only limited degradation data, with a prediction accuracy of over 85%, which meets practical application requirements.

## 1. Introduction

As industrial systems become increasingly complex, the maintenance and management of large hydraulic pumps in steam turbine control systems present significant challenges [1]. A fault may result in complete system failure. Therefore, it is crucial to assess the Remaining Useful Life (RUL) of the pump beforehand and perform necessary repairs or replacements. Evaluating the RUL of hydraulic pumps with long useful life and no failure data holds great significance.

Currently, research on methods for predicting RUL can be broadly categorized into three principal approaches: physical model-based methods, data-driven methods, and hybrid methods that combine elements of both. There exist several prediction methods based on physical failure models [2,3,4,5,6,7,8], which primarily involve analyzing the failure mechanisms of components or equipment and subsequently establishing a physical failure model to predict RUL. However, for equipment operating under complex conditions, establishing an accurate physical failure model can prove challenging. Data-driven RUL prediction methods, such as artificial neural networks [9,10,11], support vector machines [12], hidden Markov models [13,14], Bayesian networks [15,16,17], and Wiener processes [18,19,20], are widely utilized. These methods primarily employ degradation data and historical fault data obtained through online monitoring. By utilizing the evolution and development law of intelligent algorithms or probability density functions, a performance degradation model is established to achieve equipment RUL prediction, leading to significantly improved prediction accuracy. The hybrid prediction method refers to a combination of a physical failure model and data-driven methods [21] or a combination of different data-driven methods [22]. Yang et al. [23] employed the Gamma process of random parameters and Copula function to establish a binary correlation degradation model of the system. They introduced Bayesian theory and demonstrated the strong applicability of the non-conjugate prior distribution hypothesis of random parameters in engineering practice through case analysis. These methods model the evolution of performance degradation variables in the equipment within a stochastic framework and provide an expression of the remaining lifespan distribution in the form of a probability distribution. This not only allows for the estimation of the remaining lifespan but also enables the description of prediction uncertainty (such as variance, confidence intervals, and other uncertainty quantification metrics). Such uncertainty quantification is crucial for scientific decision-making related to maintenance, replacement, and logistics support, making statistical data-driven methods a hot topic and mainstream approach in this field both domestically and abroad.

The commonly used stochastic models in statistical data-driven methods for predicting the remaining lifespan include stochastic coefficient regression models, Wiener processes, Gamma processes, inverse Gaussian processes, and so on [24]. The Wiener process is a type of diffusion process driven by Brownian motion, which is suitable for characterizing non-monotonic degradation processes. It provides more flexibility in modeling degradation measurement data and has been used for degradation modeling and remaining lifespan prediction in various types of equipment. For instance, Li [25] proposed a sequential Bayesian updating method for the Wiener process model to predict the remaining lifespan, where the Bayesian estimate of the previous random drift parameter was used as the prior for the next update. In this way, the current Bayesian estimate of the random drift parameter depends on the entire degradation measurement history up to the current time, thus addressing the issue of relying solely on the current degradation measurement. Joseph and Yu [26] assumed that there exists a transformation method that can convert nonlinear degradation features into linear features, and then used the Wiener process for degradation modeling. Si [27] proposed a nonlinear degradation model that transforms a diffusion process with nonlinear drift coefficients that have a constant threshold into a linear model with a variable threshold. This approach can significantly improve the estimation of the remaining lifespan. Zhang [28] proposed a degradation model with flexible random effects and a parameter estimation framework based on the Expectation Maximization (EM) algorithm. The main idea of these methods is to use stochastic processes to describe the evolution of the equipment performance degradation variables. By estimating the model parameters based on monitoring data, the probability distribution of the time to reach the failure threshold of the established stochastic degradation process can be solved, which enables the prediction of the remaining lifespan. Therefore, these methods have a natural advantage in quantifying the uncertainty of remaining lifespan prediction. Ma et al. [29] analyzed typical failure modes of hydraulic pumps and established a relevant life model. Using failure data obtained from an accelerated test, RUL prediction of an aviation hydraulic pump was conducted. The oil return flow was identified as an appropriate parameter to reflect the axial piston pump, and the Kalman filter method was utilized to estimate parameters, with the effectiveness of the method verified through experiments.

Based on the literature analysis above, it can be observed that there has been a considerable amount of research on degradation modeling and remaining lifespan prediction for randomly degrading equipment, especially in the area of statistical data-driven methods. However, it should be noted that the existing research mainly focuses on modeling the evolution trajectory of equipment performance degradation variables that have a certain trend and generally adopts a fixed degradation model function form. In practice, the selection of the degradation model function form itself is a challenge, especially when the chosen model function form is not suitable. It is difficult to achieve effective calibration of the degradation model solely through updating the model parameters, which can affect the accuracy of remaining lifespan prediction. Hydraulic pump is a kind of special equipment with long service life and a lack of full life cycle operation data. Although there are failure data obtained through historical fault data and accelerated experiments, considering the cost and test cycle, there is a lack of complete degradation cycles of the sample. Therefore, the early calibrated model is often difficult to adapt to the accurate prediction of the remaining service life of the hydraulic pump in the later period of service. Therefore, in order to meet the actual demand of residual service life prediction for randomly degraded equipment, it is necessary to develop a method that accurately predicts the residual service life by using the dynamic degradation distribution model and its parameters based on the previous limited degradation data.

This paper proposes an RUL prediction method for hydraulic pumps based on limited degradation data. The method utilizes only a small amount of real-time monitoring data to construct a limited dataset, with volumetric efficiency as the degradation indicator. The optimal degradation curve is established using the degradation trajectory model, and the optimal probability distribution model is selected via K-S test. The above process is repeated to optimize the degradation model and update parameters in different performance degradation stages of the hydraulic pump, providing quantification of the prediction uncertainty and enabling accurate online prediction of the hydraulic pump’s RUL.

## 2. Methods

### 2.1. Degradation Indicator

This study focuses on the PVH74QIC-RSM-IS-10-C25-31 swash plate variable piston pump as the research object, using volumetric efficiency as the criterion to evaluate the pump’s operational status. According to 6.2.2 of JB/T7043-2006, as shown in Table 1, the volumetric efficiency of the piston pump should not decrease by more than 3 percentage points of the standard value after the RUL test. When the volumetric efficiency of the piston pump drops to 88%, it is deemed to be invalid.

The volumetric efficiency of the pump represents the ratio of actual flow to theoretical flow. In JB/T7043-2006, the calculation formula for the volumetric efficiency of an axial piston pump is given by Equation (1):

(1) $$\eta_{v}=\frac{V_{2,e}}{V_{2,v}}=\frac{q_{v2,e}/n_{e}}{q_{v2,i}/n_{i}}\times 100\%$$

where $\eta_{v}$ is the volume efficiency of the axial piston pump, $V_{2,\varepsilon }$ is the displacement at test pressure (in units of mL/r), $V_{2,i}$ is the no-load displacement (in units of mL/r), $q_{v2,\varepsilon }$ is the output flow at test pressure (in units of L/min), $q_{v2,i}$ is the output flow at no-load pressure (in units of L/min), $n_{\varepsilon }$ is the rotational speed at test pressure (in units of r/min), and $n_{i}$ is the rotational speed at no-load pressure (in units of r/min).

Equation (1) shows that the volumetric efficiency is calculated using online monitoring parameters such as flow rate and rotational speed, and it is used as the degradation data $z\left(t\right)$. The degradation curve $y\left(t\right)$ characterizes the degradation trend, and $\varepsilon \left(t\right)$ represents the random fluctuation during operation. Therefore, the degradation model is expressed as Equation (2):

(2) $$z\left(t\right)=y\left(t\right)+\varepsilon \left(t\right)$$

### 2.2. Degradation Path Model

In the case of degradation data (volumetric efficiency), selecting an appropriate degradation trajectory is crucial. Curve fitting is utilized to investigate the relationship between degradation and RUL, making it easier to reveal the degradation behavior of equipment. As the degradation model is unknown, this paper performs curve fitting using exponential, Fourier, linear, and quadratic models. The formula for curve fitting is given by Equation (3):

(3) $$\left\{\begin{array}{l} y=ae^{bx}\\ y=a+b\cos\left(\omega x\right)+c\sin\left(\omega x\right)\\ y=wx+b\\ y=ax^{2}+bx+c \end{array}\right.$$

The merits and demerits of curve fitting are evaluated using the following three indicators to determine the optimal degradation curve:

(1) Residual sum of squares (SSE): SSE represents the error between the curve fitting value and the test value, indicating the degree of model fitting. A smaller SSE value indicates better model fitting, as it reflects a closer match between the fitting method and the real test result. This leads to more successful data prediction.
(2) Root Mean Square Error (RMSE): RMSE represents the standard deviation between the fitting value and the test value. A smaller RMSE value indicates a better fitting effect, as it reflects a closer match between the fitting result and the test value. When the RMSE is closer to 0, it means that the fitting result is closer to the test value, indicating a better fitting effect.
(3) R-square (also known as the coefficient of determination): R-squared is a statistical measure that represents the square of the correlation coefficient between the measured data (test value) and the fitted value. Its value ranges from 0 to 1. A value closer to 1 indicates a better fitting effect, implying that the curve fitting method is more accurate. In other words, R-squared measures the proportion of the variation in the dependent variable (test value) that is explained by the independent variable (fitted value).

### 2.3. Probabilistic Distribution Model

In this study, the Weibull distribution model, extreme value distribution model, and lognormal distribution model were selected as probability distribution model candidates for random effects. The best probability distribution model, which is most consistent with random effects, was determined using the Kolmogorov-Smirnov (K-S) test.

#### 2.3.1. Weibull Distribution Model

The Weibull distribution is a theoretical basis for reliability analysis and life testing [30]. It has a strong fitting ability for test data and is widely used in data processing for various life tests due to the ease of deducing its distribution parameters by probability value. Additionally, the Weibull distribution can be transformed into other distributions, such as the exponential, Rayleigh, and normal distributions, by changing its parameters, which is another important reason for its wide application. The probability density function of the Weibull distribution is given by Equation (4):

(4) $$f(t)=\left\{\begin{array}{ll} \frac{m}{\eta }{\left(\frac{t-\gamma }{\eta }\right)}^{m-1}e^{-{\left(\frac{t-\gamma }{\eta }\right)}^{m}}, & \;t\geq \gamma \\ 0, & t<\gamma \end{array}\right.$$

where t is temporal random variable, m>0 is the shape parameter, η>0 is the scale parameter, and γ>0 is the position parameter. It is called a two-parameter Weibull distribution when γ=0.

#### 2.3.2. Model of Extreme Distribution

The extreme value distribution is a probability distribution that describes the distribution of the maximum (or minimum) value in probability theory. It represents the probability density distribution that each maximum value selected from many independent values should follow [31]. The probability density function of the extreme value distribution is given by Equation (5):

(5) $$f(t)=1-e^{-e\left(\frac{t-\mu }{\sigma }\right)}$$

where t is a temporal random variable, μ is the position parameter, and σ is the scale parameter.

#### 2.3.3. Lognormal Distribution Model

The lognormal distribution is a probability distribution that describes a random variable whose logarithm follows a normal distribution [32]. In the short term, the lognormal distribution is very similar to the normal distribution. However, in the long term, the lognormal distribution has a more pronounced upward trend. The probability density function of the lognormal distribution is given by Equation (6):

(6) $$f\left(t\right)=\left\{\begin{array}{ll} \frac{1}{t\sqrt{2\pi }}e^{\left[-\frac{{\left(\ln t-\mu \right)}^{2}}{2\sigma^{2}}\right]}, & t>0\\ 0, & t\leq 0 \end{array}\right.$$

where t is a temporal random variable, μ is the mean value, σ is the variance.

### 2.4. K-S Test

The K-S test is a commonly used method to assess whether an empirical distribution follows a theoretical distribution or to compare whether two empirical distributions have significant differences [33]. The K-S test compares the distribution model of the test data with a pre-specified distribution model. If the difference between the two distribution model parameters is small, the sample is considered to follow a specific distribution. The K-S test method is simpler and more accurate than other test methods.

The key test parameters of the K-S test are D and *p*, where D is an indicator variable: accepting the null hypothesis that the data follow the theoretical distribution (D ≤ critical value) or rejecting the null hypothesis that the data do not follow the theoretical distribution (D > critical value). *p* is the significance probability, usually set at 0.05, which determines whether to accept or reject the null hypothesis. The greater the *p*-value, the better the probability distribution model, and the closer it is to the reality.

### 2.5. RUL Prediction

The reliability $R\left(t\right)$ of a product represents the probability that the product can guarantee its normal operation at time t, that is, the time that the product can still work normally when the probability of the product working normally is above a certain level R, as shown in Equation (7):

(7) $$P\left(\xi >t\right)=R\left(t\right)=R$$

A hydraulic pump volume efficiency reduction to 88% is considered to indicate that the end of its working life has been reached. In other words, when the degradation curve $y\left(t\right)$ first reaches the pre-set failure threshold $y_{c}$, the remaining useful life (RUL) is determined. Assuming that the failure probability density function is $f\left(y|t\right)$, the degradation failure distribution function can be expressed as Equation (8):

(8) $$F\left(t\right)=\int_{0}^{t}f\left(y|t\right)dx$$

The reliability function can be expressed as Equation (9):

(9) $$R\left(t|y_{c}\right)=1-F\left(t|y_{c}\right)$$

**Figure 1:**
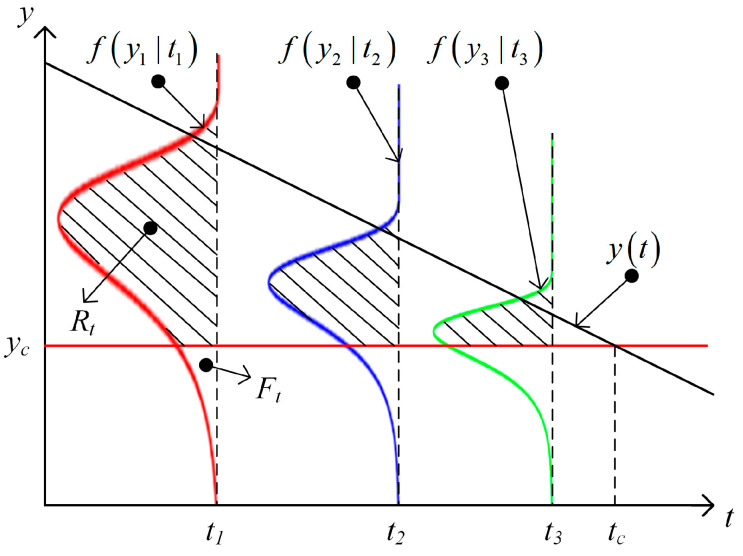
The degradation curve with probability distribution function.

In Figure 1, the distribution function of the density function $f\left(y|t\right)$ and the product life T of the degradation curve $y\left(t\right)$ at time t are shown. The shaded area represents the reliability $R_{t}$ at time t. The failure threshold $y_{c}$ is then introduced into the degradation curve at the time $t_{c}$ on the abscissa under the specified reliability. The remaining useful life value under the current reliability can be obtained, and the uncertainty of the prediction can be quantified.

### 2.6. RUL Prediction Method

The RUL prediction method for hydraulic pumps based on degradation data proposed in this study is as follows:

(1) Determining the failure criteria of hydraulic pumps and investigating the degradation laws of degradation data over time.
(2) Fitting the degradation curve, calculating the residual data, and eliminating outliers.
(3) To achieve this, different fitting methods such as exponential fitting and linear fitting are used to characterize the optimal function that matches the degradation data over time. The best fitting method is selected through evaluation index. Additionally, a random variable is determined to characterize the random fluctuations of the degradation data around the degradation curve.
(4) Based on the theory of probability and statistical analysis, the parameter identification of random variables in different probability distribution models is performed. The optimal probability distribution model is determined using the K-S test to quantify the uncertainty.
(5) The failure time of the hydraulic pump is calculated by determining the given reliability probability of a given time t.

**Figure 2:**
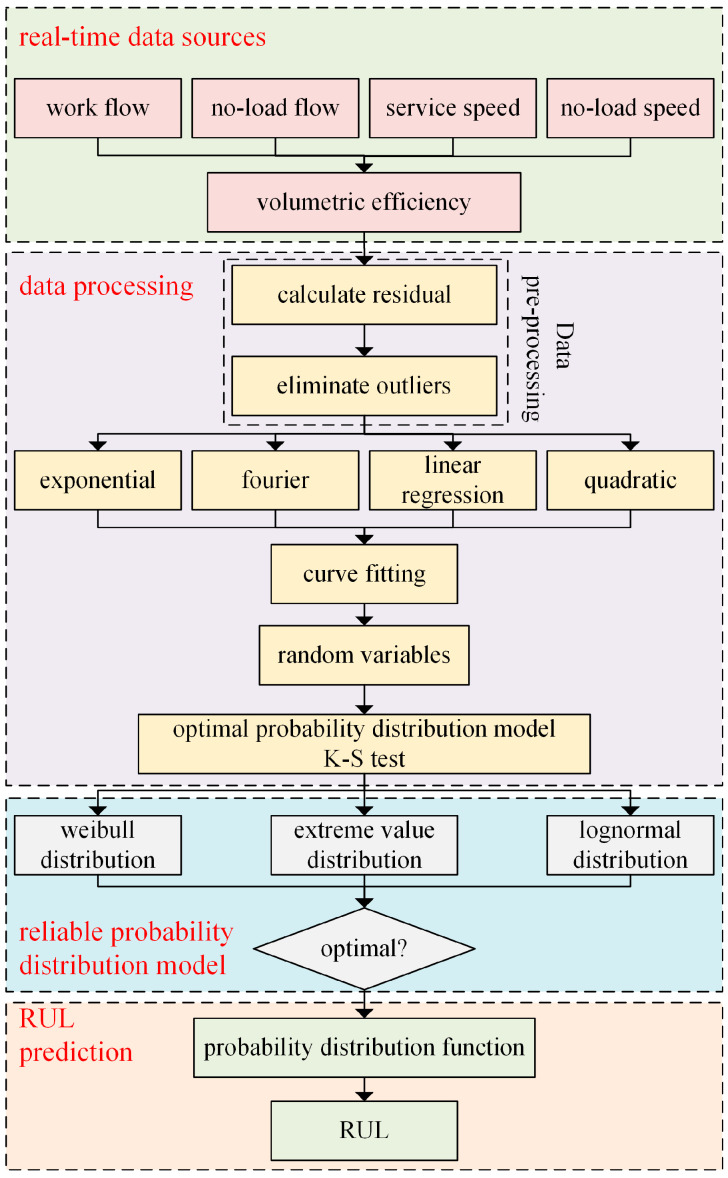
Prediction flow chart.

The prediction process is illustrated in Figure 2. As the prediction time increases and more data is accumulated, the prediction results become more accurate.

## 3. Test System

**Figure 3:**
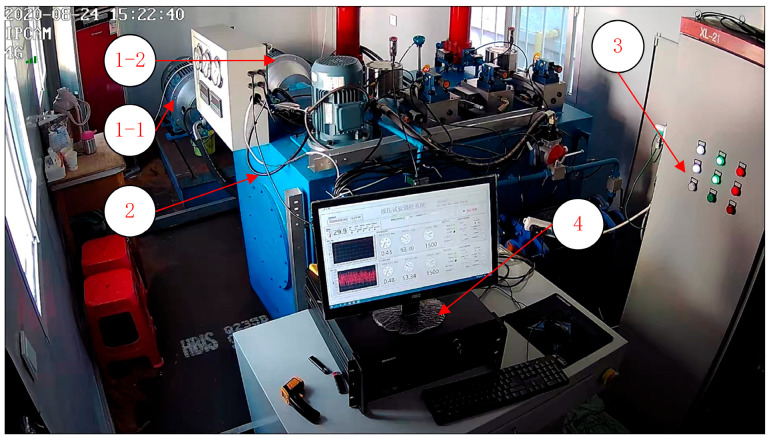
Piston pump RUL test bench. 1-1—used pump for testing, degraded state, 1-2—new pump for testing, initial state, 2—control bench, 3—PLC control cabinet, 4—control computer.

Based on the data required in Equation (1), a remaining life test bench for the plunger pump is constructed as shown in Figure 3. The test bench system utilizes the Siemens S7-1200 series PLC as the control core, which is responsible for signal acquisition and equipment control. The collected data is transmitted to the monitoring computer in real-time. The monitoring computer is responsible for displaying current data, operating controls, recording data, and other functions.

**Figure 4:**
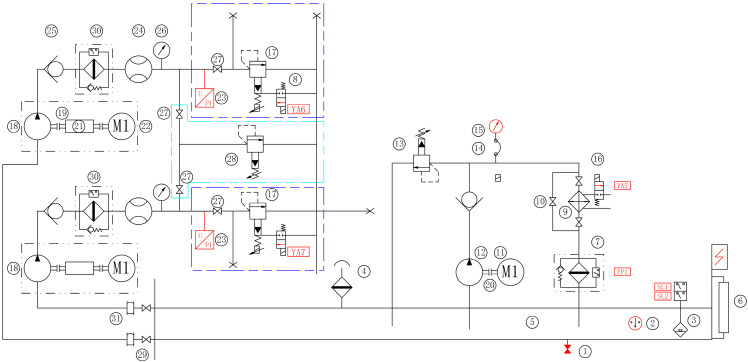
RUL test bench schematic. 1—oil drain plug, 2—temperature sensor, 3—Liquid level control relay, 4—air filter, 5—oil tank, 6—Liquid Level Thermometer, 7—return filter, 8—magnetic exchange valve, 9—water-cooler, 10—high pressure ball valve, 11—motor, 12—vane pump, 13—overflow valve, 14—Pressure measuring hose, 15—pressure gauge, 16—solenoid water valve, 17—proportional relief valve, 18—hydraulic pump, 19—main motor-pump coupling, 20—Sub-motor-pump coupling, 21—velocimeter, 22—main motor, 23—pressure transducer, 24—flow transducer, 25—check valve, 26—Shockproof pressure gauge, 27—high pressure ball valve, 28—high-pressure safety valve, 29—butterfly damper, 30—high-pressure filter, 31—expansion joints.

The basic principle of the test bench is illustrated in Figure 4. Each motor-pump unit independently controls the operating conditions through an electro-hydraulic proportional relief valve. A spare safety relief valve of the same specification is added through a parallel oil circuit composed of ball valves to achieve emergency treatment. The input signal of the proportional relief valve is adjusted to meet the demand of pressure change under different working conditions. Additionally, an independent circulating cooling and filtration system is designed to ensure that the oil temperature remains stable during the test, with the stable range is controlled at ±5 °C. This consideration is made due to the fact that all energy is lost at the relief valve port and the heating phenomenon is serious. Finally, real-time high-precision measurement of the hydraulic pump speed and output flow is performed, and data acquisition and processing are completed to study the remaining service life of the hydraulic pump based on good operating stability.

**Figure 5:**
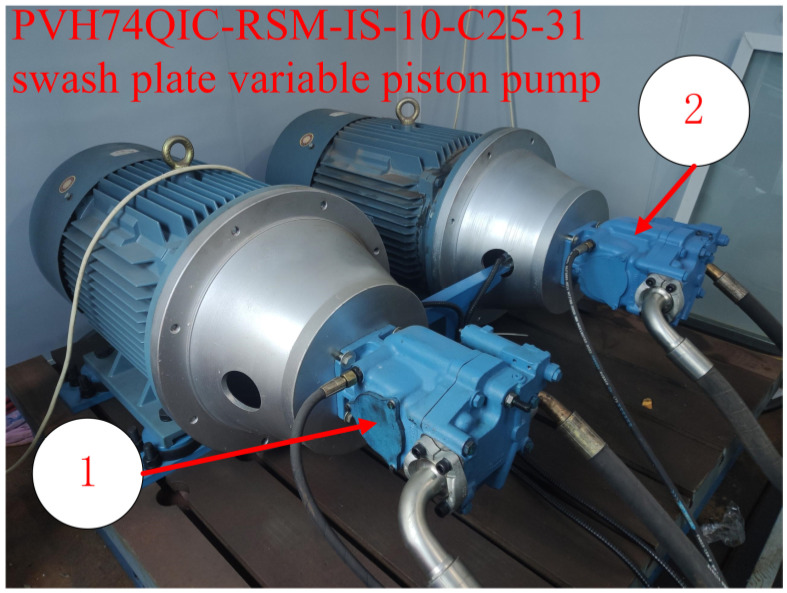
The measured piston pumps. 1—used pump for testing, degraded state, 2—new pump for testing, initial state.

During the test, the automatic circulating cooling system ensures that the test oil temperature remains within the working oil temperature range of the equipment, which is 40~55 °C. The pump to be tested is shown in Figure 5, with the new pump on the left and the old pump on the right. As piston pumps have a long service life, the early data of the new pump does not change significantly. Therefore, the test data of the old pump with an obvious degradation trend is used for analysis.

## 4. Discussion

### 4.1. Feature Extraction

The displacement, speed, and flow data were collected through the online arrangement of sensors, and the collected data was processed and calculated to obtain volumetric efficiency. The data were recorded once per minute. Due to the large amount of data, the table only lists the average volume efficiency calculated every four days. The data for the first 120 days is shown in Table 2.

Based on the data in Table 1, the data points of volumetric efficiency are plotted on a coordinate system. The fitting indices for exponential fitting, Fourier fitting, linear fitting, and quadratic fitting are shown in Table 3.

**Figure 6:**
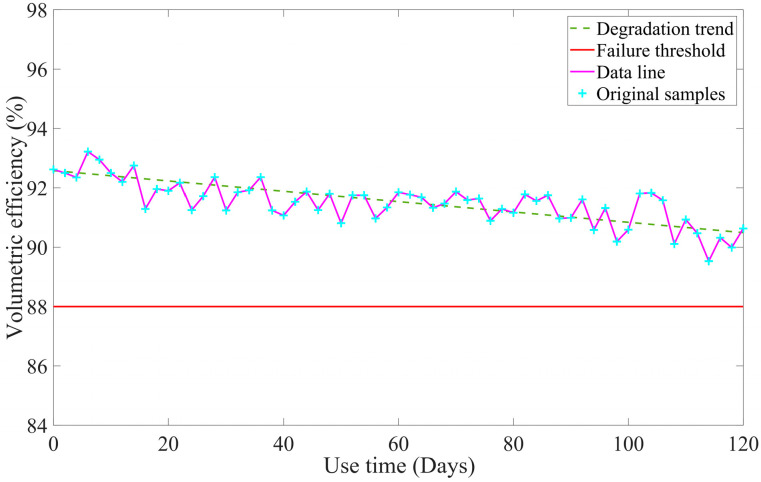
The fitting curve of volumetric efficiency.

Table 2 indicates that linear fitting is deemed to be the optimal method. The volumetric efficiency data points in the coordinate system are fitted linearly, as shown in Figure 6.

**Figure 7:**
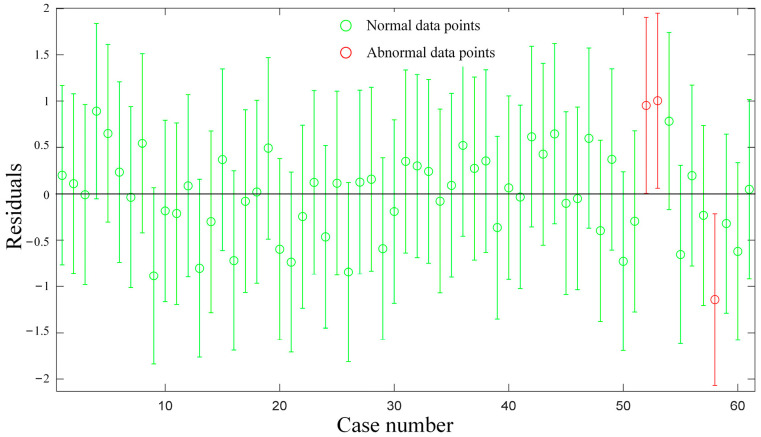
Residual analysis chart.

During the experiment, external factors may cause anomalies in the data, which can introduce errors in subsequent analysis results. These anomalous data points are referred to as data anomalies. To ensure the accuracy of the results, it is necessary to perform residual analysis on the data to eliminate any data anomaly points. The difference between the observed value and the predicted value (fitting value) is known as the residual. By analyzing the information concealed within the residual, residual analysis can be employed to determine the reliability of the data. In regression analysis, standardized residuals of the test data points that fall outside the (−2, 2) interval can be classified as data outliers at a 95% confidence level and should be directly eliminated. The results of the residual analysis are presented in Figure 7.

The expression for the fitting curve after eliminating the abnormal data points is given by Equation (10).

(10) $$\eta_{v}=-0.0174t+92.5754$$

where $\eta_{v}$ is the volumetric efficiency, t is using time.

**Figure 8:**
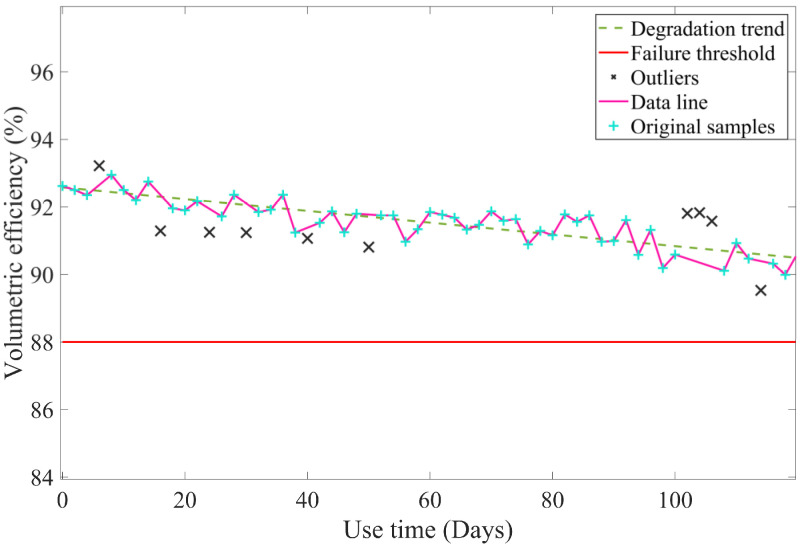
The re-fitting degradation curve of volumetric efficiency.

The degradation curve, after the elimination of outliers, is illustrated in Figure 8.

### 4.2. Modelling

The random effect primarily accounts for the random fluctuations observed in the degradation data around the degradation curve, denoted by $\varepsilon_{t}=z\left(t\right)-y\left(t\right)$. The data resulting from this effect are presented in Table 4.

**Figure 9:**
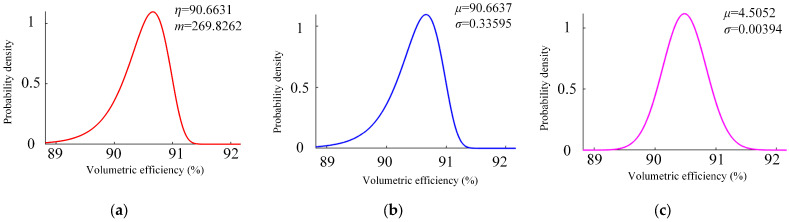
Three kinds of probability distribution model. (**a**) Weibull distribution model, (**b**) Extreme value distribution model, (**c**) Lognormal distribution model.

The data presented in Table 3 are used as random variables to construct Weibull, extreme value, and normal distributions, and the volumetric efficiency is obtained according to $\varepsilon_{t}$. The results are presented in Figure 9.

The K-S test results are shown in Table 5. The *h* values of the three models are 0, indicating that they are in line with the data. The *p* value of the lognormal distribution model in the three models is closer to 1, indicating a higher consistency. Therefore, the most consistent probability distribution model is the lognormal distribution.

**Figure 10:**
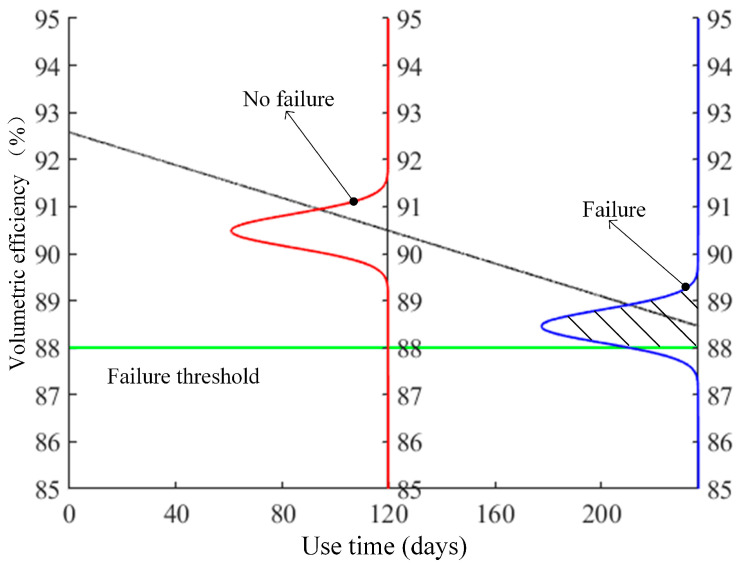
Remaining useful life calculation schematic diagram.

The probability distribution curve is shifted along the direction of the fitting curve until the area above the failure reference line corresponds to a given probability of 90%. At this point, the probability distribution curve stops moving, and the corresponding abscissa on the curve is 236.0443 days, as shown in Figure 10. Therefore, the piston pump can be used for at least 116 days with a reliability probability of 90%.

The trend of the RUL with the change of reliability probability can be obtained from the diagram, which fully demonstrates the intuition of the proposed method and the accuracy of relying on probability and statistics theory. As the amount of monitoring data continues to increase, the prediction accuracy also improves.

### 4.3. Results and Analysis

As the hydraulic pump life test has a long duration, it may not reach the failure state during the test, making it difficult to verify the accuracy of the prediction results. To address the challenge of obtaining long-term and complete prior sample data, this study proposes a method of changing the failure reference value to verify the accuracy of the proposed method.

Using the available 120 day test data as a basis, the failure reference value is set as the volumetric efficiency observed on the 120th day, at which point the hydraulic pump is considered to have failed. Five observation points are selected at the 20th, 40th, 60th, 80th, and 100th days. The proposed method is used to predict the RUL at each of these observation points, and the accuracy of the method is verified by comparing the deviation between the predicted results and the actual results.

The deviation between the predicted RUL and the actual value is denoted as E, and the calculation formula for the deviation is given by Equation (11).

(11) $$E=\frac{\left|R_{0}-R\right|}{R_{0}}\times 100\%$$

where $R_{0}$ is actual RUL, R is predicted RUL.

**Figure 11:**
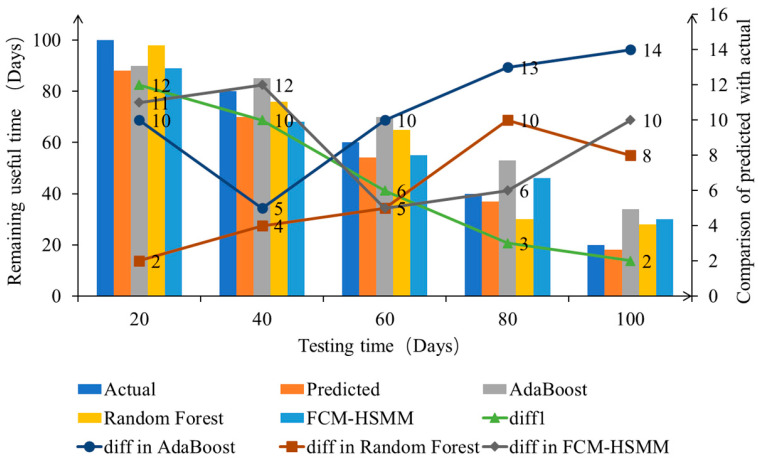
Comparison of actual RUL and predicted RUL.

Figure 11 presents the difference and deviation between the predicted and actual RUL.

Figure 11 shows that compared to some RUL prediction methods, the initial prediction accuracy of the proposed method is not superior at the beginning of observation under the training condition of limited data. However, as the observation time increases, the prediction error of the proposed method shows a monotonically decreasing trend. The prediction fluctuation gradually decreases when the reliability is set to 90%, with a deviation within 15%. When the set reliability is 85%, the remaining life is predicted again using the same comparison method as above, and the deviation is still within 15%.

Based on the comparative analysis presented above, it can be inferred that the method proposed in this paper achieves high prediction accuracy and low computational complexity while using only limited degradation data. This method enables accurate online prediction of hydraulic pumps.

## 5. Conclusions

In this study, we propose a method for predicting the RUL of hydraulic pumps based on limited degradation data, which enables accurate online prediction of hydraulic pumps that have long service lives, are difficult to shut down, and have no failure data. The main conclusions of this study are as follows:

(1) The RUL method proposed in this study constructs a degradation trajectory model using volumetric efficiency as the performance degradation indicator. The method achieves a prediction accuracy of over 85% while only using limited degradation data. Compared with traditional AdaBoost, Random Forest, and FCM-HSMM, the prediction error of the proposed method shows a monotonically decreasing trend, and the prediction fluctuation gradually decreases. Additionally, the computational complexity is low, ensuring both the accuracy and real-time performance of the prediction.
(2) This study proposes an evaluation method for curve fitting and probability distribution models for unknown degradation models. The method selects the best degradation curve and probability distribution model, effectively revealing the degradation law of hydraulic pumps and quantifying the uncertainty of random effect.
(3) This study proposes a verification method based on changing failure threshold, which enables the test verification of hydraulic pumps with long service lives and no failure data by assuming a failure threshold.

## Figures and Tables

**Table 1:** Volumetric efficiency and total efficiency of axial piston pump from 6.2.2 of JB/T7043-2006.

	Swash Plate Piston Pump			Inclined Shaft Piston Pump	
nominal displacement (mL/r)	2.5	10≤V<25	25≤V≤500	10≤V<25	25≤V≤500
volumetric efficiency $\left(\%\right)$	≥80	≥91	≥92	≥94	≥95
overall efficiency $\left(\%\right)$	≥75	≥86	≥87	≥84	≥85

**Table 2:** Volume efficiency for the first 120 days.

Use Time (Days)	Volumetric Efficiency (%)	Use Time (Days)	Volumetric Efficiency (%)
0	92.62	64	91.68
4	92.35	68	91.47
8	92.95	72	91.59
12	92.2	76	90.89
16	91.29	80	91.16
20	91.9	84	91.56
24	91.25	88	90.97
28	92.36	92	91.61
32	91.85	96	91.32
36	92.36	100	90.59
40	91.07	104	91.83
44	91.87	108	90.11
48	91.8	112	90.47
52	91.75	116	90.32
56	90.97	120	90.63
60	91.85		

**Table 3:** Fit index.

	SSE	RMSE	R-Square
exponential fitting	14.5	0.4958	0.5507
Fourier fitting	24.8	0.6596	0.2301
linear fitting	13.7	0.4558	0.5907
quadratic fitting	14.5	0.5	0.5507

**Table 4:** Vertical distance calculation results.

Use Time (Days)	$\varepsilon_{t}$	Use Time (Days)	$\varepsilon_{t}$
0	−0.0446	64	−0.2187
4	0.1558	68	−0.0783
8	−0.5139	72	−0.2679
12	0.1665	76	0.3625
16	/	80	0.0228
20	0.3273	84	−0.4468
24	/	88	0.0736
28	−0.2720	92	−0.6361
32	0.1684	96	−0.4157
36	−0.4113	100	0.2447
40	/	104	/
44	−0.0605	108	0.5854
48	−0.0601	112	0.1558
52	−0.0798	116	0.2362
56	0.6306	120	−0.1435
60	−0.3190		

**Table 5:** K-S test results.

	Weibull	Extreme Value	Lognormal
*h*	0	0	0
*p*	0.80416	0.7971	0.96173

## Data Availability

The data presented in this study are available on request from the corresponding author. The data are not publicly available due to the data confidentiality requirements of the company where the testing equipment is located.
